# Subtotal petrosectomy in cochlear implantation: Gruppo Otologico experience after 348 cases

**DOI:** 10.1007/s00405-025-09431-8

**Published:** 2025-05-15

**Authors:** Luca Morelli, Sachin K. Damam, Hailu Yilala M., G. Fancello, M. Ferraro, A. Caruso, M. Sanna

**Affiliations:** 1 Department of Neurotology and Skull Base Surgery, Gruppo Otologico, Piacenza, Rome, Italy; 2 Audiology Unit, Department of Clinical Sciences and Community Health, Department of Specialistic Surgical Sciences, State University of Milano, Fondazione IRCCS Cà Granda, Ospedale Maggiore Policlinico, Milano, Italy; 3 Vijaya Health Center & Rudh Hearing & Speech Clinic, Bangalore, India; 4 Department of ORL-HNS, Addis Ababa University, Addis Ababa, Ethiopia; 5 ENT Department, Azienda Ospedaliera di Legnago – Mater Salutis, Verona, Italy

**Keywords:** Cochlear implants, Subtotal petrosectomy, Cochlear malformations, Anatomic variations, Cochlear ossification, Chronic otitis media

## Abstract

**Objective:**

To discuss indications (unfavorable conditions), surgical steps, complications, and follow-up of subtotal petrosectomy (STP) in cochlear implantation based on our experience of 348 cases. Anatomical variations associated with or without cochlear malformations and differences between electrode insertions were included in our analysis.

**Materials and methods:**

A retrospective case study was done in Gruppo Otologico (Piacenza, Italy), a quaternary referral center. Among 1002 cases that underwent subtotal petrosectomy, 348 were selected for cochlear implantation in the same setting. The study period was from 2004 to 2019. These patients’ clinical and radiological follow-up ranged from 2 months to 180 months. Data were inspected, cleaned, and analyzed by SPSS software.

**Results:**

The selected group’s mean age was 57.236 years, including 178 male and 170 female patients. The follow-up period lasted up to 108.65 months. Out of 348 cases, 8 were children (under the age of 18 years old). Inclusion criteria to be eligible for cochlear implantation in the same setting of subtotal petrosectomy was a preoperative pure tone average (PTA) of more than 90 decibels associated with low speech discrimination ranging from 0% in most cases up to 50% in the minority. 329 patients had a complete electrode insertion intra-operatively. Minor complications were observed in 6 cases. These include one case of implant extrusion due to a middle ear infection leading to labyrinthitis, three cases of post-auricular fistula causing one device failure, one subcutaneous CSF collection, and one subcutaneous seroma collection.

**Conclusion:**

Although in most cases, standardized trans mastoid facial recess technique for cochlear implantation is ideal as the surgical risks are minimal, in complicated cases such as concomitant chronic otitis media, prior canal wall-down cases, radical cavities, or inner ear abnormalities with high risk of cerebrospinal fluid leak subtotal petrosectomy should be the first choice of management with complete disease clearance. Close clinical and radiological follow-up is therefore mandatory. Single-stage implantation is preferred to staging the procedure unless one is unsure of disease clearance.

## Introduction

Nowadays cochlear implant (CI) represents the primary solution for hearing rehabilitation in people with severe to profound hearing loss. Combining the cochlear implantation in the same setting of subtotal petrosectomy (STP) can be adopted in situations depending on the indications with a low risk of complications.

Based on our experience and supported by literature it is well-established that, in the vast majority of cases, the standardized trans mastoid facial recess technique for cochlear implantation is ideal as the surgical risks appear to be minimal [1]. Some cases, however, can represent a challenge and force the surgeon to prefer a more radical approach. Concomitant chronic otitis media, prior canal wall-down cases, radical cavities, temporal bone trauma violating the otic capsule, cochlear ossification, or inner ear abnormalities with a high risk of cerebrospinal fluid leak in the same setting of cochlear implantation [1–5] in our experience require a more radical solution leading to performing a subtotal petrosectomy as the most effective choice in management with complete disease clearance.

Although the surgical technique of mastoid obliteration was first introduced by Rambo [2] in the year 1958 with the terminology of primary closure of radical mastoidectomy, Ugo Fisch introduced the term “subtotal petrosectomy” in 1965 [4, 8] describing STP as the complete exenteration of all pneumatic tracts in the temporal bone, isolating middle ear cleft from the external environment and nasopharynx by removing mastoid cells, mucosa, and epithelium and closing the external auditory canal (EAC) in a blind sac closure with plugging of the eustachian tube.

First described for treating temporal bone tumors and skull base pathology, it was later combined with CI by Sanna (unpublished data) and other authors [3–5]. Issing et al. and Bendet et al. in 1998 [9, 10] recommended STP as the preferred method for cochlear implantation in ears affected by chronic otitis media (COM).

The primary aim of the present article is to discuss indications, surgical steps, complications, and follow-up of subtotal petrosectomy in cochlear implantation. Anatomical variations associated with cochlear malformations & differences between electrode insertions were included in our analysis.

## Objective

Based on our experience of 348 cases:

1. To discuss the indication and surgical steps in simultaneous STP and CI.
2. To determine postoperative outcomes, complications, and long-term follow-up results of subtotal petrosectomy in cochlear implantation.

### Study design

A retrospective case study from 2004 to 2019 in Gruppo Otologico, Piacenza, Italy: a quaternary skull base center.

## Materials and methods

Among 1002 cases that underwent subtotal petrosectomy, 348 were selected for cochlear implantation in the same setting between 2004 and 2019 at Gruppo Otologico. 178 males and 170 females, from 2 to 79 years including 8 pediatric patients. All surgeries were performed in a single stage. The subjects provided informed consent, and the hospital committee ethically approved the study.

Patients had a full oto-neurologic, audiologic, and radiological assessment (CT and MRI) before the indication of subtotal petrosectomy with cochlear implantation and evaluation of cochlear lumen patency. Preoperative work-up consists of comprehensive counseling (including vaccination recommendations), facial nerve motility examination, audiological assessment (pure tone audiometry, auditory brain response ABR, and speech discrimination test), imaging (Temporal Bone CT without contrast and brain MRI with gadolinium).

Data were collected from the patient’s medical records and included demographic information, clinical management, intraoperative findings, postoperative complications, and clinical and radiologic follow-up findings. Follow-up was defined as the time from surgery to the most recent clinical evaluation.

**Fig. 1:**
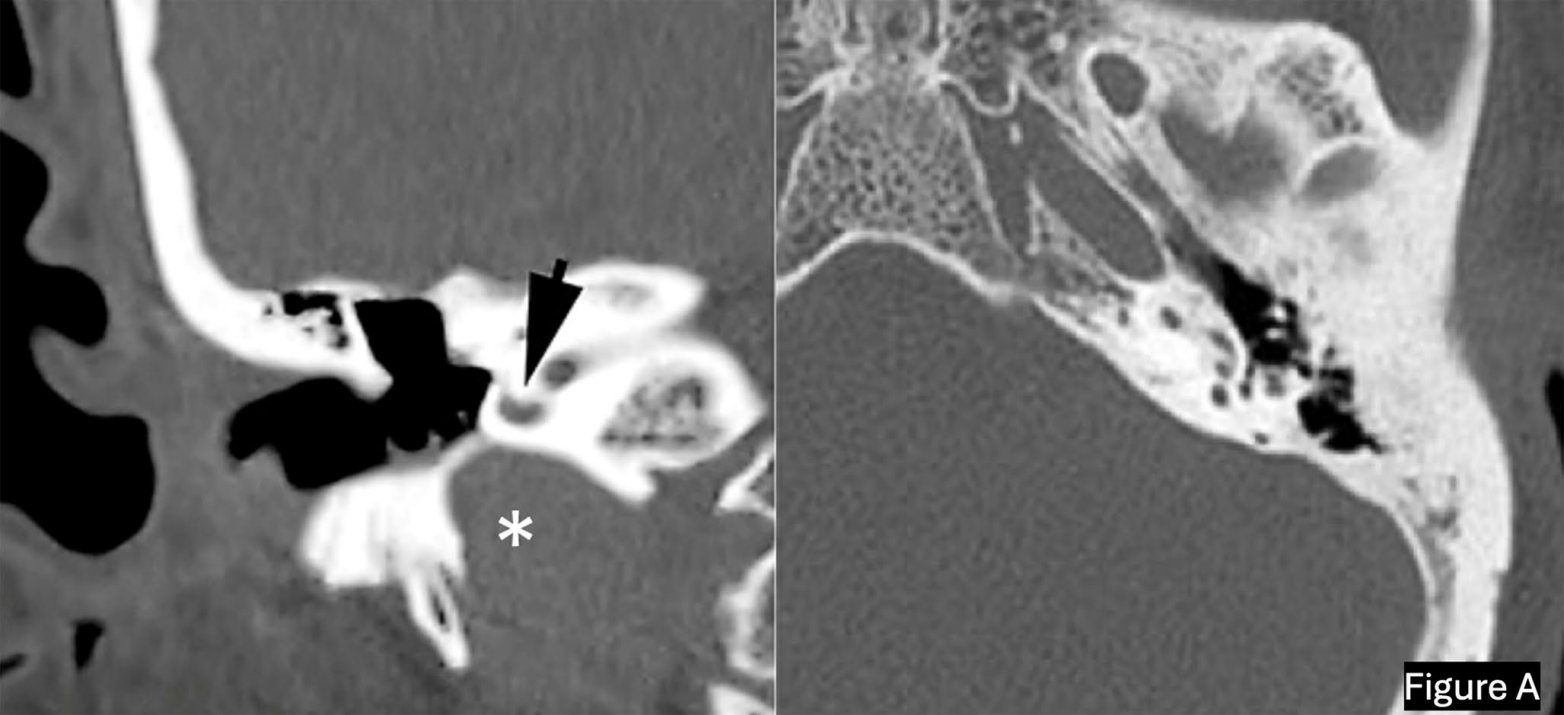
In the left pre-operative high-resolution CT (HRCT) scan is visible an example of a high jugular bulb (white asterix) in direct contact with the basal turn of the cochlea (black arrow) in a patient with a history of tympanoplasty and a current indication for CI; on the right HRCT scan, left temporal bone ossification of the round window, basal and apical turn of the cochlea secondary to otosclerosis

The decision to perform a simultaneous subtotal petrosectomy with cochlear implantation instead of the standardized trans mastoid facial recess technique, which is the ideal technique in the vast majority of cases, was based on the patient’s history, physical findings, radiological peculiarities (Fig. 1) and history of previous middle ear surgery or recalcitrant chronic otitis media.

**Fig. 2:**
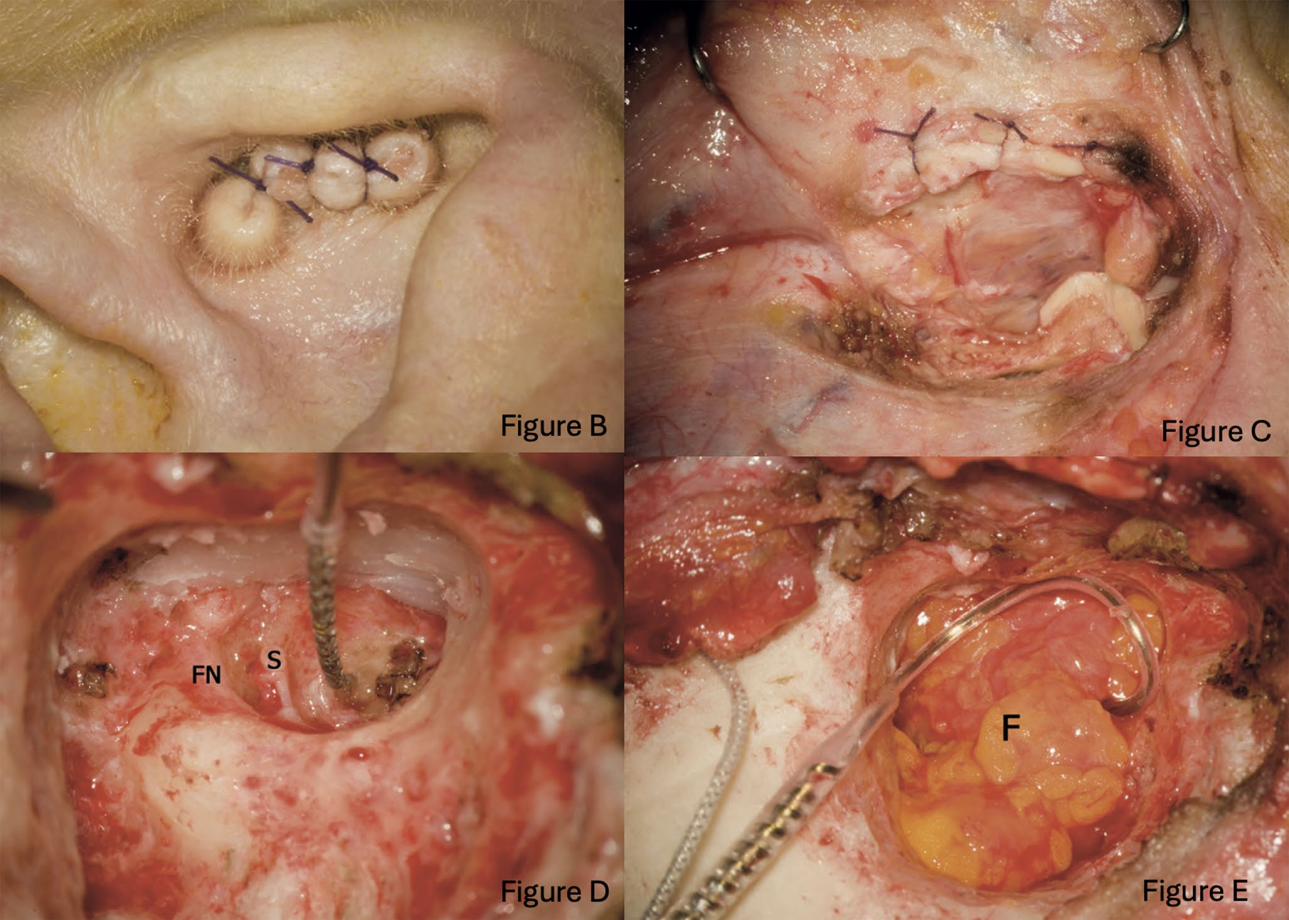
Peculiar subtotal petrosectomy in cochlear implantation’s steps in our center

Subtotal petrosectomy in cochlear implantation steps in our center were: **1**. blind sac closure of the external auditory canal (figure B & C) followed by **2**. exenteration of middle ear and mastoid air cells, including peri-sigmoid, peri-labyrinthine, peri-facial, and hypotympanic; **3**. removal of the malleus and incus; in the eventuality the stapes is not left intact by the pathology it is necessary to proceed also with the removal of the residual ossicular chain possibly leaving the platina in place; **4**. drilling receiver-stimulator bed and fixed with sutures or bone wax, **5**. cochlear implantation with the closure of the Eustachian tube with periosteal soft tissue and temporalis muscle (Fig. 2D) and **6**. obliteration of the cavity with abdominal fat previously soaked in an antibiotic solution containing Rifampicin (Fig. 2) [18, 21].

In our study, standard antibiotic prophylaxis consisted of Ceftriaxone 2 g given intravenous from induction to 3 days postoperatively. Patients were routinely discharged on the fourth postoperative day and continued the same antibiotic through the intramuscular route for 6 more days until 10 days postoperatively.

The most important initial step of subtotal petrosectomy is the blind sac closure of the external auditory canal EAC, a second layer must be created by the posterior reflection of the tragal cartilage, which helps to reinforce the closure. In canal wall-down cavities, the blind sac closure could present more difficulties due to the lack of residual tissue, making of paramount importance the use of cartilage to secure the closure. The facial ridge has to be lowered until the mastoid segment of the fallopian canal is visible to offer the best exposure to the round window niche. For this reason, removing the bone anterior to the facial nerve is crucial. Another important challenge performing this surgery stands about the decision regarding the removal or sparing the mastoid tip. The original technique requires removal of the tip however sometimes could be left intact especially in small sclerotic bones as seen on Figure D. The cavity should be completely devoid of mucosa to prevent the recurrence of the disease.

**Fig. 3:**
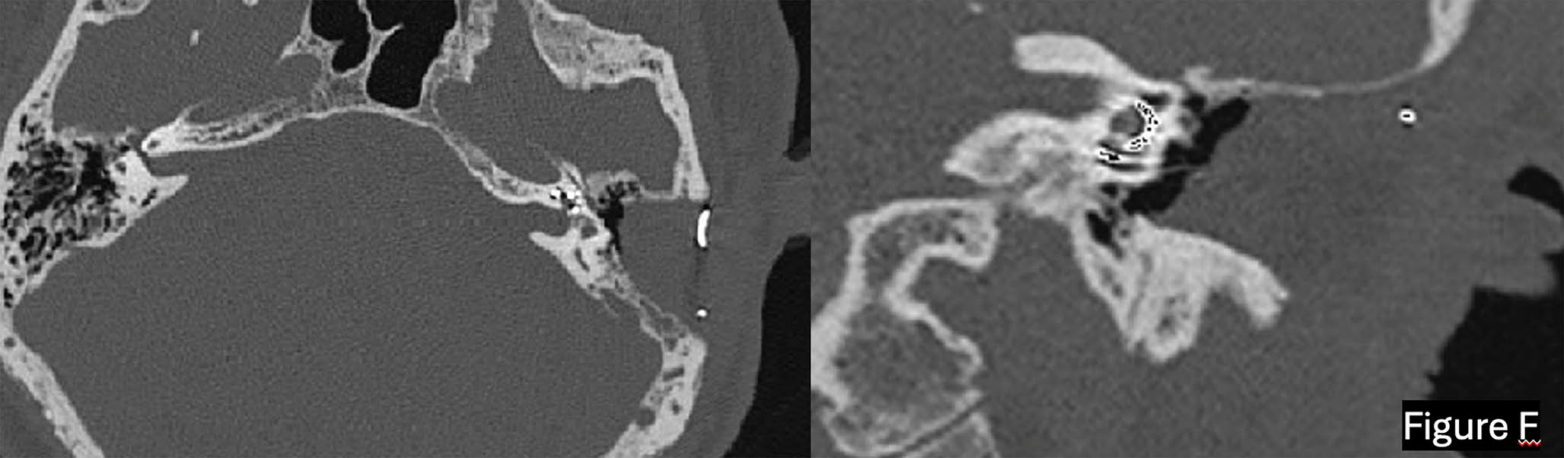
In this post-operative high-resolution CT (HRCT) scan is visible an example of the electrode array placed correctly

When the implant is fully in place, the electrode array must be accommodated on the cavity’s medial wall and held in place by fat and bone wax, decreasing the likelihood of extrusion in the area by proximity to the subcutaneous layer (Fig. 3). Delving deeper into Fig. 3F, in the axial projection on the left side of the picture, a large aeration of the cavity is visible, along with multiple uncleared air cells in the posterior part. This is due to an incomplete exenteration of the mastoid cells, a condition that frequently occurs when petrosectomy is performed for anatomical indications rather than invasive pathology. In such cases, the surgeon may opt for a less radical exenteration to preserve a smaller mastoid cavity.

The standard approach through the round window was accessible in the vast majority of cases (318). Still, when the round window was not accessible, a cochleostomy was performed in 22 patients, in 5 cases was required to enlarge the cochleostomy and drill the basal turn of the Cochlea and insertion through scala vestibuli in other 3 cases.

## Results

**Table 1:**
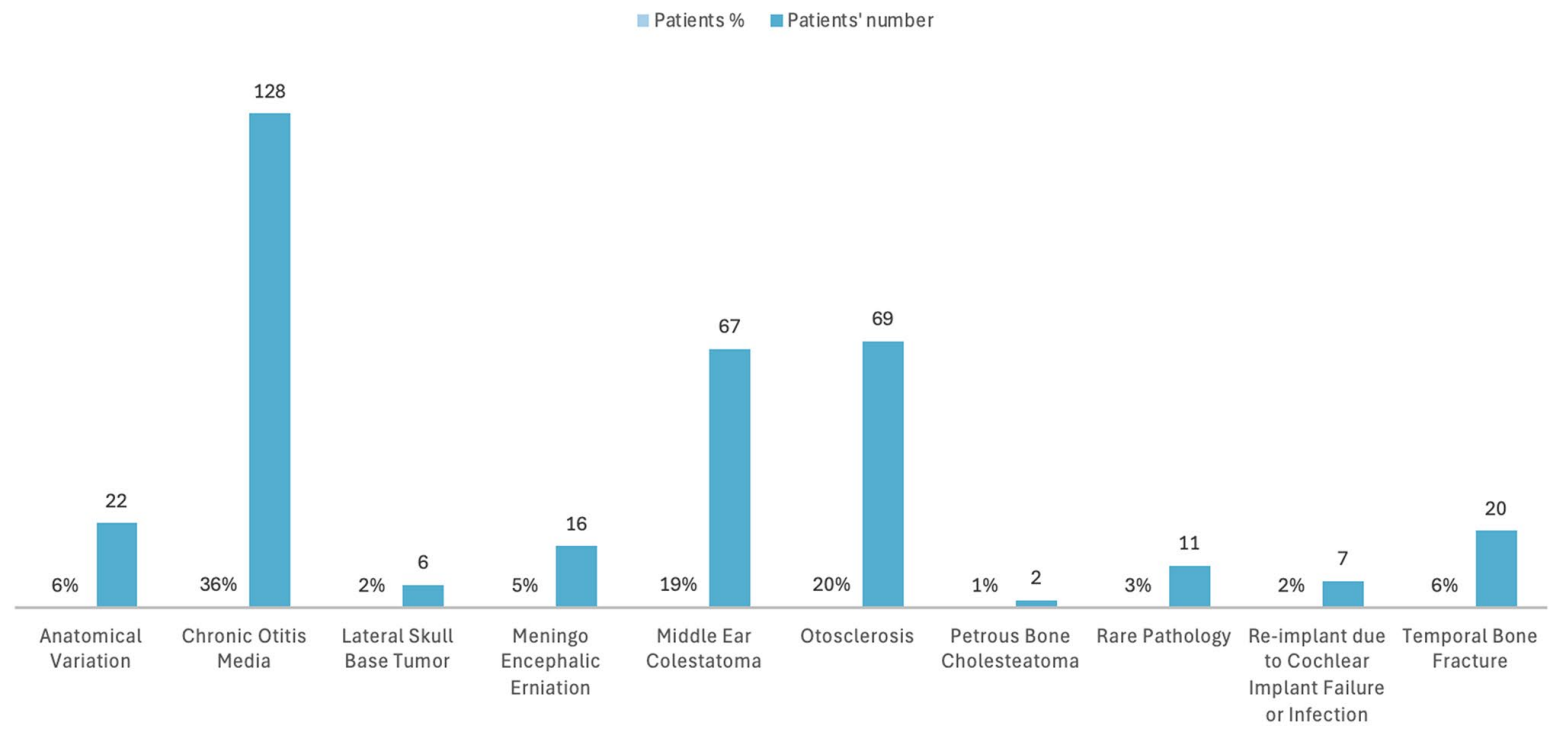
Indications which led Us to choose subtotal petrosectomy were multiple, such as anatomical variation in 22 cases (6%), chronic otitis media in 128 cases (36%), lateral skull base tumor in 6 patients (2%), meningo encephalic erniation in 16 (5%), presence of middle ear Colestatoma were found in 67 cases (19%), otosclerosis in 69 cases (20%), petrous bone cholesteatoma in 2 cases (under 1%), subtotal petrosectomies due to a re-implant in 6 cases (2%) and lastly due to a Temporal bone fracture in 20 cases (6%)

Among the 1002 cases of subtotal petrosectomy, the decision to perform cochlear implantation in the same setting was made in 348 cases (indications which led us to choose subtotal petrosectomy are summarized in Tables 1, 2, 3 and 4). The selected group’s mean age was 57.236 years, including 178 male and 170 female patients. The follow-up period lasted up to 108.65 months. Out of 348 cases, 8 were children (under the age of 18 years old).

**Table 2:**
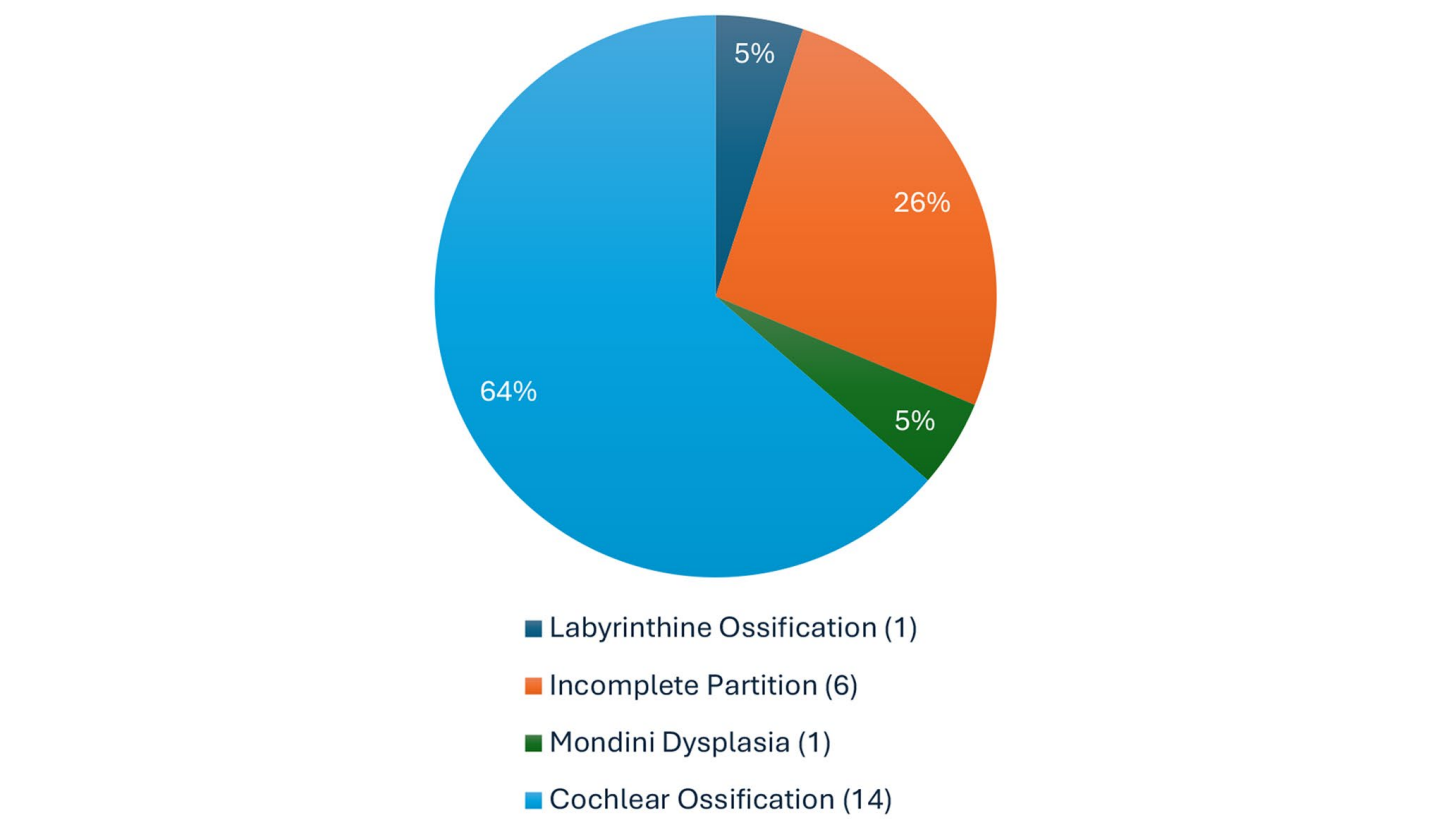
Among anatomical variation, there were 5% of patients with Labirintine ossification, 26% with an incomplete cochlear partition (highlighted in table 4), 5% had Mondini dysplasia and 63% cochlear ossification (Table 3)

**Table 3:**
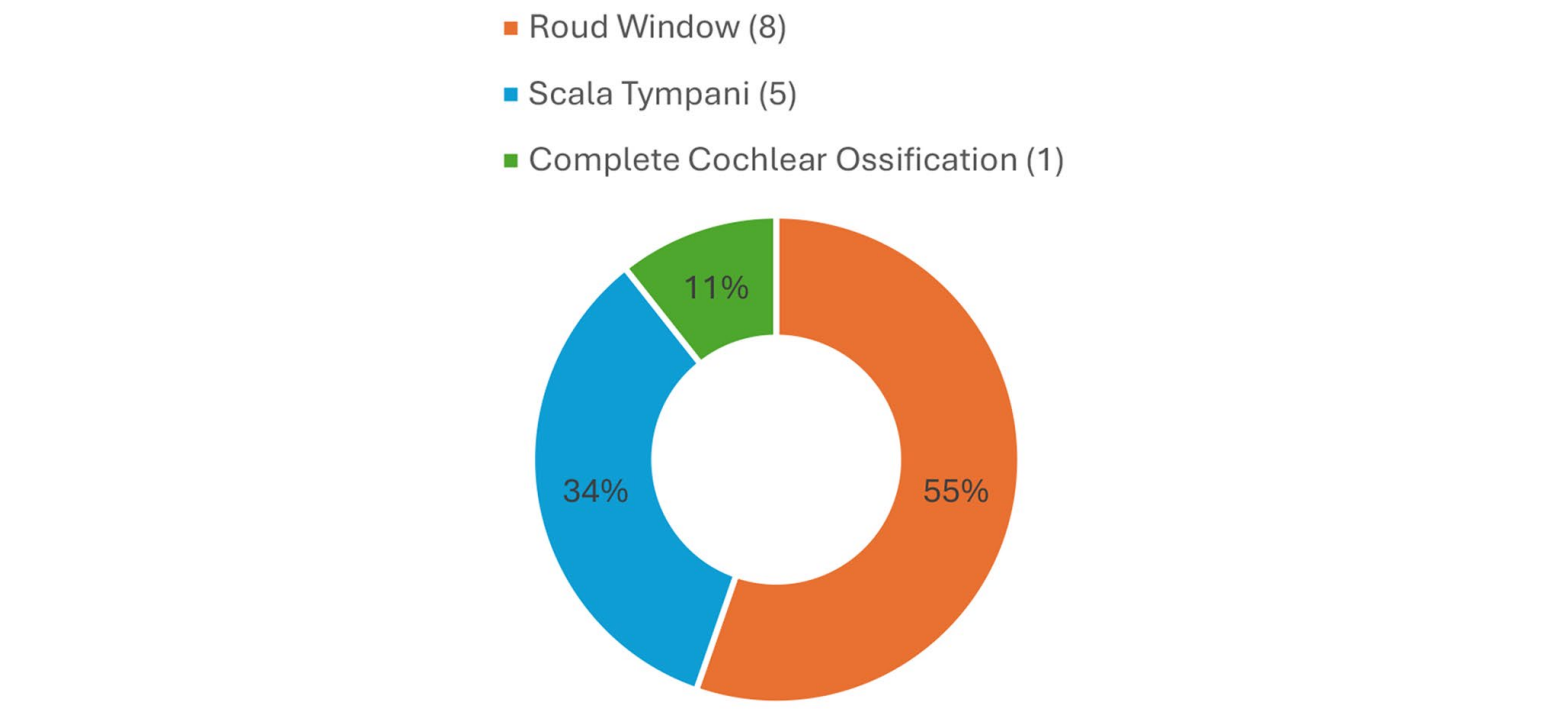
Cochlear ossification

**Table 4:**
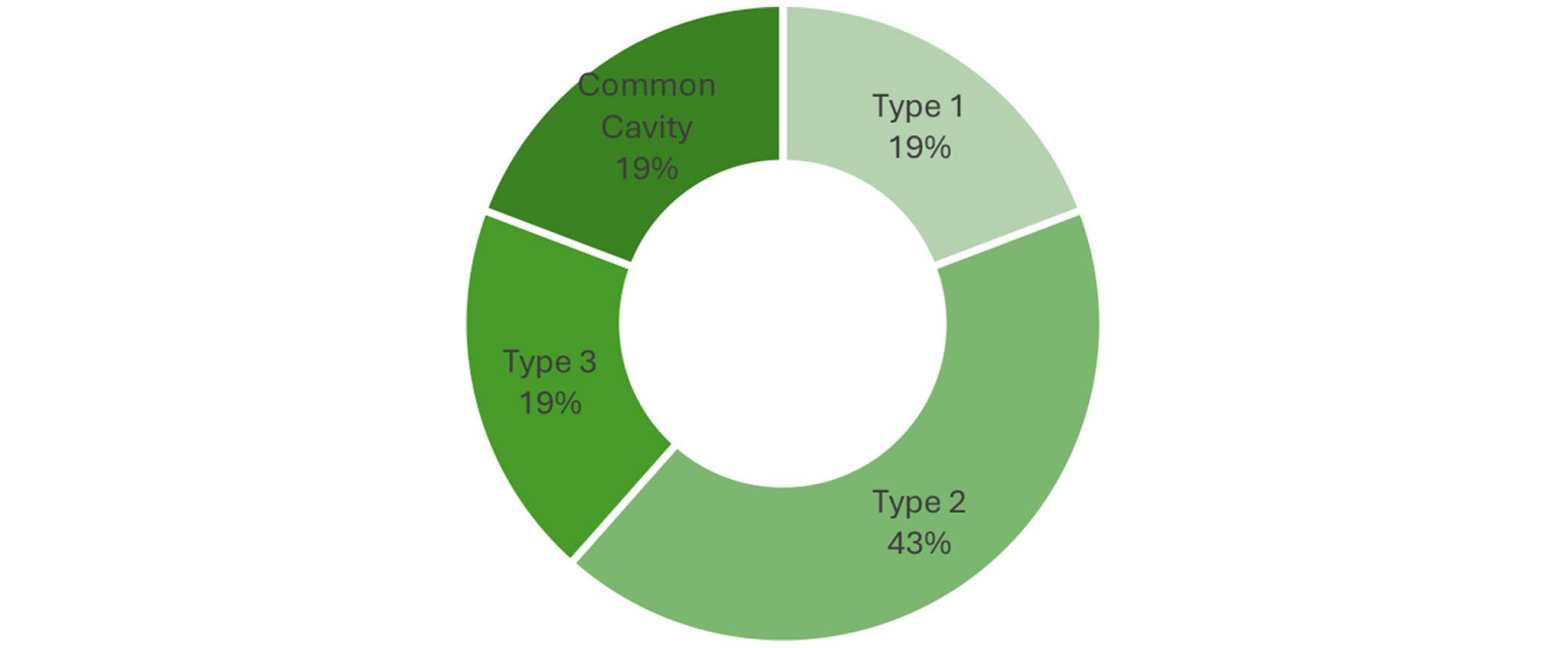
Incomplete cochlear partition

Inclusion criteria to be eligible for cochlear implantation in the same setting of subtotal petrosectomy was a preoperative pure tone average (PTA) of more than 90 decibels associated with low speech discrimination ranging from 0% in most cases up to 50% in the minority. 329 patients had a complete electrode insertion intra-operatively. All active electrodes were fully inserted into 329 cochleas except in 19 cases, the maximum number of electrodes out-ranged from one to four.

Our case series follow-up period ranged from 2 to 248 months with a mean period of 108.65 months. Although diffusion-weighted non-EPI MRI is recommended worldwide as the gold standard evaluation for residual cholesteatoma, we were forced to choose a Temporal Bone CT scan for our radiological follow-up due to the artifacts caused by MRI in the presence of a cochlear implant.

Following a first CT right after surgery to check the right electrode insertion, initial radiological follow-up was done at 6 months and then continued at 1, 3, 5, and 10 years postoperatively to detect residual cholesteatoma. No residual cholesteatoma or recurrence was highlighted in our cohort of patients. The capability of CT to detect cholesteatoma recurrency increases with the presence of abdominal fat which creates an ideal interface for differentiation from cholesteatoma [19].

Cochlear Implants were activated between 20- and 30-days following surgery, and in only one case we did notice no active electrodes. This was a revision case in which a middle ear infection was worsened by labyrinthitis, resulting in full modiolus destruction. Despite complete electrode insertion, the patient had no auditory sensation. CSF gusher was encountered intra-operative during implantation in 4 instances i.e. 1 in otosclerosis, 1 in Pendred syndrome, 1 in Mondini’s dysplasia, and 1 in an ordinary case. Post-operative complications among our cases included one cochlear implant extrusion due to a middle ear infection leading to labyrinthitis,1 three post-auricular fistula, one device failure, one subcutaneous CSF collection, and a subcutaneous seroma collection.

Pre-operative mean hearing threshold weighted four-frequency average (PTA [0.5 kHz + 1 kHz + 2 kHz + 4 kHz]/4) on pure tone audiometry was 114 dB HL (standard deviation, SD, 11dB HL) and average Speech Discrimination Score of 12% (s.d. 4%) at 96 dB HL compared to 48 dB HL (s.d. 8 dB HL) and SDS of 54% (s.d. 14%) after 6 months from cochlear implant activation in the ear selected to implant (PTA *p* = 0.004, SDS *p* = 0.008). These data show no significant differences from the previous data we published about auditory outcomes after a cochlear implant through the standardized trans mastoid facial recess technique [22]. When compared with data in the literature, these audiological results can be attributed to the heterogeneity of the etiologies that indicated the need for subtotal petrosectomy.

## Discussion

Subtotal petrosectomy (STP), with obliteration of the middle ear and mastoid and closure of the external auditory canal (EAC), is an effective solution in mastoid and middle ear diseases that are chronic and recurrent, leave behind a large surgical cavity, exposing vital structures like the dura, carotid, jugular bulb or cerebrospinal fluid (CSF), with no realistic chance of reconstruction of the conductive apparatus [7]. 

Subtotal petrosectomy decreases the chance of disease recurrence and crafts a closed and protected space from the external environment, significantly lowering the risk of infection and, consequently, array extrusion. Extensive drilling of cell tracts is required to create bare bones for better adherence of abdominal fat to cavity walls and in order to prevent mucosal granulomas caused by remaining inflammatory tissue [3]. Lastly, complete control of all the anatomical landmarks grants a safer surgical technique raising the chances of a proper and full insertion even in complex circumstances.

Multiple studies have been published in the literature about subtotal petrosectomy and simultaneous cochlear implantation (as represented in Table 5) [13]. Among the largest cohort of patients analyzed, Ruben Polo et al. in 2016 reported 110 cases with multiple details on indications, long-term follow-up, and different complications encountered [19]. Lyuntenski et al. in 2016, reported 212 cases with no details on follow-up, staging the procedures in 94.4% of cases for cochlear implantation, and reported recurrent cholesteatoma in 2.3% of cases, device extrusion in 0.5% of patients [14]. Although, according to some authors [6, 9, 15], staging strategy can reduce the risk of meningitis due to the risk of inserting an electrode in a potentially contaminated field and also reduce the risk of residual cholesteatoma, in our cohort of patients treated with a single stage procedure we experimented no higher percentage of complications in agreement with other authors [5], especially no residual cholesteatoma or recurrence was highlighted in our cohort of patients through radiological follow up with Temporal Bone CT scan.

**Table 5:** Cornerstone literature about subtotal petrosectomy and simultaneous cochlear implantation

Study	No. of Cases	Follow-Up Duration	Indications	Failures	Complication or Follow-DP Pathology
Bendet et al. 1998 [10]	5	No details	COM, Cholesteatoma, Temporal bone fracture.	No details	No details
Free et al. 2013 [1]	32	18 months	COM, Cholesteatoma, Temporal bone fracture, otosclerosis.	Electrode extrusion in 1 case.	1 case of Subcutaneous CSF collection.
Baranano et al. 2013 [16]	36	No details	COM, Cholesteatoma.	Explanation 8.3%, Electrode extrusion 2.8%,	Abscessas in 8.3% of cases and EAC Fistule in 2.8%.
Aristegui I. et al. 2022 [23]	92	9 months to 12.5 years.	COM, Cholesteatoma, Otosclerosis, Meniere Disease, Vestibular schwannoma. Inner Ear Malformation	Device Failure in 3.26% of cases.	No patients with cholesteatoma recurrence.
Szymanski et al. 2016 [17]	19	8 months to 10 years.	COM and Osteoradio-necrosis	Nil	Failure of EAC closure in 5.2% patients.
Ruben polo et al. 2016 [19]	110	12 months to 108 months	COM, Cholesteatoma, Temporal bone fracture, otosclerosis.	Device failure in one case and cochlear implant extrusion in 4 (0.03%).	1 case of subcutaneous CSF leak, 1 seroma collection and 1 patient suffered transient facial palsy (0.009%).
Golda Grinblat et al. 2020 [20]	39	85 months	COM and Cholesteatoma	Device explant in 1 case.	Post-auricular fistula in 3 cases and cavity infection in1 case.
Present study	348	2 months to 15 years (mean follow up of 108.65 months)	COM, Cholesteatoma, Temporal bone fracture, otosclerosis, Meniere Disease, Vestibular schwannoma. Inner Ear Malformation.	Device failurein 1 case (0,002%) and extrusion in 4 patients (0,01%).	Post-auricolar fistula in 3 cases, layrinthitis in 1 case, 1 case of subcutaneous CSF leak, 1 patient with subcutaneous seroma collection.

In our study group, most patients had more than one reason that led us to choose this specific approach. Under these conditions, subtotal petrosectomy gives great control of the whole middle ear and mastoid structures, simplifying the management of anatomical peculiarities encountered during the surgery, as documented in this paper, such as anatomical variations of the jugular bulb, sigmoid sinus, and middle fossa dura. In our hands, the single-stage procedure stands as the best management choice to implant an electrode with the eradication of the existing pathology instead of staging the procedure. In case of doubt about the total removal of the cholesteatoma or middle ear infection, a staged procedure combined with cochlear implantation after 6 to 12 months is highly recommended [18]. However, it is important to keep in mind the staged strategy can significantly delay the hearing rehabilitation process.

Some authors [1, 11, 12] utilize musculo-periosteal or temporalis muscle flaps with or without fat grafts to obliterate the cavity in these patients. The use of abdominal fat grafts using fat strips to eliminate dead space in temporal bone is a routine practice at our institution, including for trans-labyrinthine and other lateral skull base surgeries in over 5000 cases. Though there is a reduction in fat volume at the end of one year, the rest of the fat preserves its properties well over time.

Linthicum [12] studied the long-term histopathological fate of mastoid obliteration tissues and concluded that fat retained volume and consistency over time despite slightly increased fibrous septations, whereas muscle lost volume but remained as a hyaline cover over mastoid bone, potentially promoting healing.

No statistically significant difference in wound healing and revision surgeries was observed between the use of the fat alone, the rotation of temporalis muscle over fat, and the use of alloplastic materials in a large series of patients. Despite past surgery-induced meatal tissue loss, none of the patients in this study required pedicle flap closure or reinforcement.

As previously reported in the results section, we proceed to examine also the auditory outcome of the study group after subtotal petrosectomy and simultaneous cochlear implant compared with the outcome after a cochlear implant through the standardized trans mastoid facial recess technique. Pre-operative mean hearing threshold weighted four-frequency average on pure tone audiometry in our group was 114 dB HL (s.d. 11dB HL) with an average Speech Discrimination Score of 12% (s.d. 4%) at 96 dB HL compared to 48 dB HL (s.d. 8 dB HL) and SDS of 54% (s.d. 14%) after 6 months from cochlear implant activation in the ear selected to implant (PTA *p* = 0.004, SDS *p* = 0.008) showing no significant differences from the previous data we published about auditory outcome after a cochlear implant through the standardized trans mastoid facial recess technique [22]. 

These data confirm that subtotal petrosectomy with simultaneous cochlear implantation can be considered just as a surgical choice preferable to the standardized trans mastoid facial recess technique, not affecting the auditory outcome, in cases that require a more radical solution with complete disease clearance such as concomitant chronic otitis media, prior canal wall-down cases, radical cavities, temporal bone trauma violating the otic capsule, or inner ear abnormalities with a high risk of cerebrospinal fluid leak in the same setting of cochlear implantation [23]. 

## Conclusion

To the best of our knowledge, this paper describes the largest cohort of 348 cases of simultaneous cochlear implantation in subtotal petrosectomies in literature.

Cochlear Implantation in subtotal petrosectomy in a single stage is an ideal choice of management with complete disease clearance in different pathologies which lowers the risk of chronic ear infections, CSF leakage, and meningitis by closing off all connection with the external environment.

Complications or risks of this procedure that can be expected are infection of the abdominal fat, breakdown of the blind sac closure within the fistula of EAC, and entrapped cholesteatoma. Radiological follow-up with Temporal Bone CT scan is therefore mandatory to check for entrapped cholesteatoma and device extrusion.
